# Relevance of Prognosis Tools in Periodontitis: A Systematic Review

**DOI:** 10.3290/j.ohpd.c_2387

**Published:** 2025-12-02

**Authors:** Amélie Sarbacher, Pierre-Yves Gegout, Olivier Huck

**Affiliations:** a Amélie Sarbacher Dentist, Université de Strasbourg, Faculté de Chirurgie-dentaire, 8 rue Sainte-Elisabeth, 67000 Strasbourg, France. Study design and concept, conducting the study, writing the manuscript.; b Pierre-Yves Gegout Associate Professor, Université de Strasbourg, Faculté de Chirurgie-dentaire, 8 rue Sainte-Elisabeth, 67000 Strasbourg, France; Pôle de médecine et chirurgie bucco-dentaire, Hôpitaux Universitaires de Strasbourg, 67000, Strasbourg, France; INSERM (French National Institute of Health and Medical Research), UMR 1260, Regenerative Nanomedicine, CRBS, 1 Rue Eugène Boeckel, 67084 Strasbourg, France. Study design and concept, conducting the study, writing the manuscript.; c Olivier Huck Professor, Université de Strasbourg, Faculté de Chirurgie-dentaire, 8 rue Sainte-Elisabeth, 67000 Strasbourg, France; Pôle de médecine et chirurgie bucco-dentaire, Hôpitaux Universitaires de Strasbourg, 67000, Strasbourg, France; INSERM (French National Institute of Health and Medical Research), UMR 1260, Regenerative Nanomedicine, CRBS, 1 Rue Eugène Boeckel, 67084 Strasbourg, France. Study design and concept, conducting the study, writing the manuscript.

**Keywords:** prognosis, regression tree, tooth retention, risk factors, periodontitis

## Abstract

**Purpose:**

Tooth-level prognostic systems can be used for treatment planning and help to identify teeth at risk of being lost over time. However, several sets of criteria were proposed to estimate tooth prognosis in the context of periodontitis patients.

**Objectives:**

To identify the prognosis tools available and to evaluate their relevance in predicting periodontal-related tooth loss (TLP).

**Methods and Materials:**

An electronic search was conducted for published data in MEDLINE, EMBASE, COCHRANE up to January 2025. Reference lists of retrieved studies for full-text screening and reviews were hand-searched for potentially eligible studies.

**Results:**

In total, 1,471 records were identified from databases, and an additional 7 unique records were identified through citation searching of selected studies from the database search. After screening, 33 studies were selected for full-text review, of which 22 were included in the final selection, with 6 classical models, 11 regression-based models, 2 AI-driven prognostic models and 3 external validations. Most prognostic models confirm high precision and accuracy, fluctuating in most cases around a value of area under the curve (AUC) = 0.8.

**Conclusions:**

Patient (smoking, diabetes) and tooth-related factors (furcation involvement, increased probing depth, mobility) influence the prognosis of tooth retention in the long term. The identification of prognostic factors is of crucial importance to better predict the long-term survival of teeth and to adapt the treatment plan. All prognostic systems at the dental level showed excellent predictive capacity for the risk of tooth loss linked to periodontitis. An AI-based machine-learning algorithm will be a helpful tool for determining tooth prognosis.

Periodontitis is an inflammatory disease characterised by the progressive destruction of tooth-supporting tissues. Left untreated, severe periodontitis can lead to extensive tooth loss (TL), masticatory dysfunction, nutritional compromise, aesthetic impairment, altered speech, and low self-esteem, thus reducing overall quality of life.^29^ Studies on the natural history of periodontitis performed in Sri Lankan tea plantation workers suggested an overall increase in both prevalence and severity of the disease with advancing age and determined different disease progression rates.^2^ The 40‐year follow‐up of the same population defined the tooth mortality due to untreated periodontitis as 0.33 teeth lost every year per patient.^41^ There is evidence that some periodontal patients suffer from greater disease severity early in life or experience a higher risk for disease progression, highlighting the need for specific and individualised treatment planning.^59^ TL can generally be prevented if disease is diagnosed and treated at an early stage for both caries and periodontal disease.^15^ In addition, regular supportive periodontal therapy (SPT) appears to be important for the reduction of TL.^18,56^

In 2008, the American Academy of Periodontology (AAP) defined risk assessment ‘as the process by which qualitative or quantitative assessments are made of the likelihood for adverse events to occur as a result of exposure to specified health hazards or by the absence of beneficial influences’.^2^ Predicting whether compromised teeth can be retained over the long term is a crucial component of treatment planning.^50^ Early periodontal screening and diagnostic tools will provide early recognition of periodontal diseases and facilitate timely management of the disease to reduce tooth loss.^6,31^ Tooth removal often leads to the need for replacing teeth and the associated risks of adverse, expensive and unforeseen complications. Retaining teeth with a poor prognosis might compromise the retention of other teeth and generate substantial costs for periodontal treatment.^53^ By assigning tooth prognosis, clinicians can discriminate between low- and high-risk teeth that may be lost due to progressive, untreated periodontitis, enabling personalised treatment planning.^18^ However, assigning periodontal prognosis represents a clinical challenge and may not always be time-efficient.^20^ Computational methods, such as artificial intelligence (AI) and machine learning, are emerging to address these diagnostic and prognostic challenges. Classical regression models are commonly used to identify predictors associated with TL. Indeed, age, gender, molar teeth, periodontal pocket depth (PPD), bone loss (BL), the presence of a root canal treatment, and prosthetic treatment are factors associated with an increased risk of TL.^34,45,62^ To date, several risk assessment tools exist, but there is no single universally accepted set of criteria for establishing a case-specific periodontal prognosis.

The objective of this systematic review was to identify prognostic tools for predicting TL in periodontitis patients and to evaluate their relevance.

## MATERIALS AND METHODS

### Protocol

This study was prepared in accordance with the Preferred Reporting Items for Systematic Reviews and Meta-Analyses (PRISMA) guidelines.^35^

### Inclusion and Exclusion Selection Criteria

Prediction model studies based on a cross-sectional, case-control, prospective or retrospective design performed in the adult population were included. The predictors included any dental, medical, or social measures, regardless of how they were determined. Studies reporting the accuracy or AUC (area under the curve) of the tooth loss prediction model were included. Studies were excluded if they targeted specific populations, such as special needs patients, lacked a cohort on which model performance was evaluated, or focused on prognostic factors without the formal development of a prediction model.

### Focused Question

The focused question of this systematic review was: What are the available prognosis tools to assess tooth prognosis in periodontally compromised patients?

The CoCoPop framework has been used for this review on non-interventional studies.^28^ Given the non-interventional nature of our study, the PICO framework was not applicable.

Condition (Co): periodontitis
Context (Co): assessed in epidemiological surveys
Population (Pop): adults
Primary outcome: tooth survival/tooth loss

### Literature Search, Screening, and Selection

The published literature was searched in three electronic databases (MEDLINE via PubMed, Cochrane, CENTRAL). The keywords used in the MEDLINE search were as follows : (‘periodontal’[All Fields] OR ‘periodontally’[All Fields] OR ‘periodontically’[All Fields] OR ‘periodontics’[MeSH Terms] OR ‘periodontics’[All Fields] OR ‘periodontic’[All Fields] OR ‘periodontitis’[MeSH Terms] OR ‘periodontitis’[All Fields] AND (‘prognosis’[MeSH Terms] OR ‘prognosis’[All Fields] OR ‘prognoses’[All Fields]) AND (‘tooth loss’[MeSH Terms] OR (‘tooth’[All Fields] AND ‘loss’[All Fields]) OR ‘tooth loss’[All Fields]). The keywords used in the Cochrane search were as follows: (periodontitis OR ‘periodontal disease’) AND (‘Tooth Loss’ OR ‘Tooth Extraction’) AND (‘prognosis’). The keywords used in the BioMed Central Journals (Oral Health) search were as follows: (periodontitis OR ‘periodontal disease’) AND (‘Tooth Loss’ OR ‘Tooth Extraction’) AND (‘prognosis’). The search process, planning, and all electronic searches were conducted by one examiner (AS). In addition, the references of each included study were checked manually by two examiners (OH and P-YG) for possible additions. Literature was searched up to January 2025.

### Data Extraction

For studies that fulfilled the inclusion criteria, one author (AS) independently extracted bibliographic details regarding the patients, predictors, outcomes, and analysis, in the following stages:

Screening potentially suitable titles and abstracts against the inclusion criteria to identify potentially relevant papers, resulting in a complete database by merging studies
Screening of the full papers identified as possibly relevant from the initial screening.

### Risk of Bias Assessment

The included studies were critically appraised by two reviewers (AS and P-YG.) using Cochrane tools (ROBINS-I). The ROBINS‑I tool (Risk Of Bias In Non‑randomised Studies – of Interventions) is used to assess the risk of bias when estimating the effectiveness or safety, whether benefit or harm, of interventions in studies that did not use randomisation to assign treatments. It evaluates bias arising from confounding, participant selection, intervention classification, deviations from intended interventions, missing outcome data, outcome measurement, and the selection of the reported result. Each domain, along with the overall study, is assigned a judgement of low, moderate, serious, or critical risk of bias, providing an overall appraisal of the credibility and reliability of findings from non‑randomised intervention studies.^57^

## RESULTS

### Study Selection

**Fig 1:**
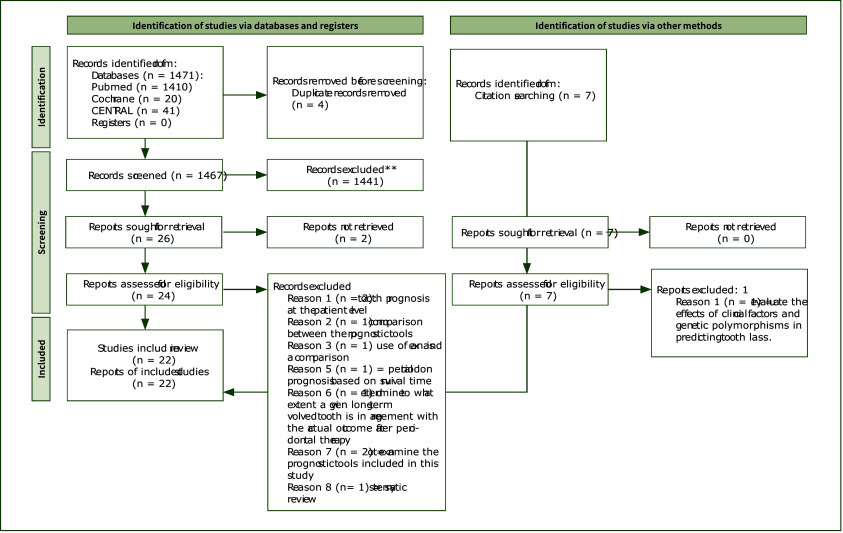
Study flow diagram.

In total, 1,471 records were identified from databases, and 7 records were identified through citation searching of selected studies. After screening, 33 studies were selected for full-text review, of which 22 were included in the final selection, with 6 classical, 11 regression-based, 2 AI-driven prognostic models, and 3 external validations (Fig 1).

### Study Characteristics

Fifteen studies were identified as model development studies.^4,5,7,11–13,17,20,24–27,33,40,47,49^ One article developed two separate models.^32^ Three studies were external validation studies.^46,51,53^ Three articles reported both development and external validation.^24,49,61^

### Risk of Bias

**Fig 2:**
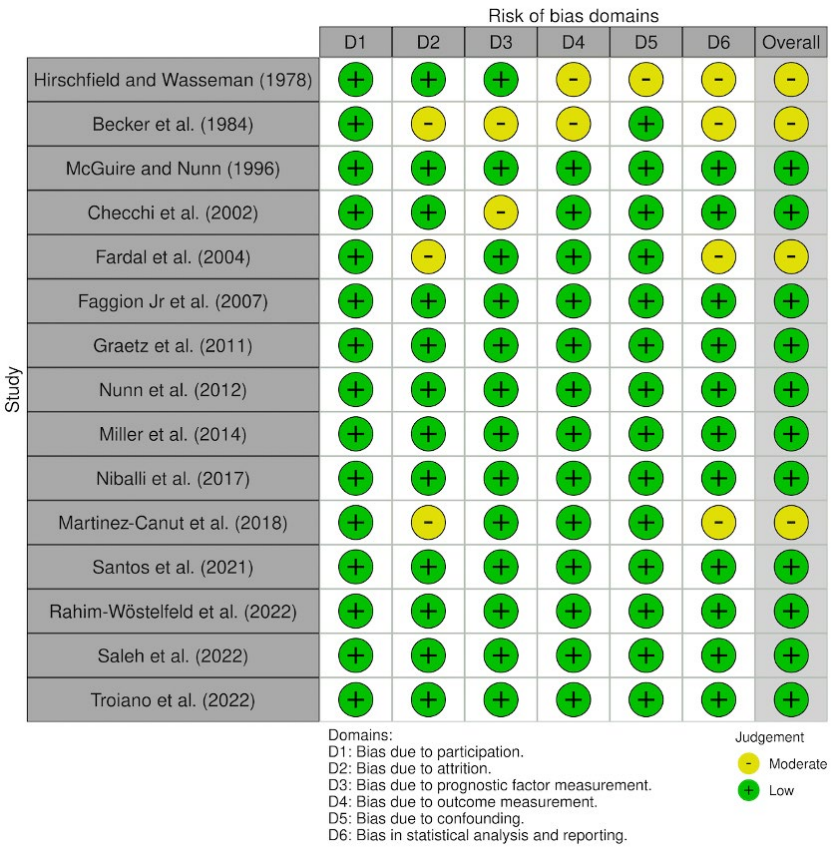
Evaluation of the risk of bias.

According to Cochrane tools (ROBINS-I), 10 of the included studies had a low risk of bias in all domains.^57^ Overall ratings are presented in Figure 2.^16^

### Data Sources, Settings, and Participant Characteristics

Data sources included mainly retrospective cohorts (19 studies).^5,7,11–13,17,24,26,27,32,33,40,46–49,51,53,61^ Eight studies were performed in a university setting^11,13,40,46,47,49,53,61^; 10 in a private practice^5,7,12,17,24,26,27,32,33,51^; and 1 combined sample populations from univer-sities and private practices.^48^ The follow-up in each study was with a minimum of 3 years, however, three studies did not report the duration of the follow-up.^4,20,25^ The number of patients considered in all studies varies between 44 and 600 patients.^5,17^

### Prognostic Tools

Prognostic tools are usually based on patient-level factors (age; diabetes status; smoking habit; periodontitis staging and grading)^43,46,53^ and/or tooth-level factors like probing pocket depth (PPD), clinical attachment level (CAL), bleeding on probing (BOP), furcation involvement (FI) and bone loss (BL) (Table 1 and Table 2).^46^

**Table 1:** Development models

Author (year)	Unit of analysis	Type of study	Setting	Key participant characteristics	Summary of follow up	Number of participants, teeth, and events in analysis	List of candidate predictors/model being validated		Tool form	Timing	Tooth survival probability	Classification System (categories)	Results	Outcome	Modelling method
Hirschfield and Wasseman (1978)^17^	Tooth	Retrospective study	Private periodontal practice	Patients who had been treated at least 15 years previously	Average duration of maintenance was 22 years and the median was 20 years	600 patients 5,666 teeth	PPD FI Mobility BL		Clinical criteria tresholds			Good Questionable	No AUC No specificity No sensitivity	Teeth lost	
Becker et al (1984)^5^	Tooth	Retrospective study	Private periodontal practice	Patients who were treated for periodontal disease and for varying reasons elected not to participate in the maintenance aspect of periodontal care.	Periods ranging from 18 to 115 months, the mean time period for patients was 3.72 years	44 patients 782 teeth	RBL PPD FI Mobility Presence/type of vertical defects Poor C/R ratio Significant root proximity Periodontal abscess		Clinical criteria tresholds			Good Questionable Hopeless	No AUC No specificity No sensitivity	Teeth lost	
McGuire and Nunn (1996)^26^	Tooth	Survey article Longitudinal study	Private periodontal practice	Periodontitis patients with at least 5 years of SPT	Mean: 9.97 years (range: 0.33–15.17 years)	100 patients2509 teeth	Age Family history Smoking Diabetes (controlled) Compliance Parafunctional habit Habit and no bite guard % bone loss Probing depth BL type Furcation Mobility C/R ratio Root form Initial prognosis		Clinical criteria tresholds	Before the treatment APT	5- to 8-year prediction accuracy of 80%overall; however, it decreased to 50% or less when the teeth with a ‘good’ prognosis were excluded	Good Fair Poor Questionable Hopeless	No AUC No specificity No sensitivity System classes to be accurate for the good and hopeless categories, with low predictive accuracy for the poor and questionable categories	Teeth lost	Cox proportional hazards model
Checchi et al (2002)^7^	Tooth	Retrospective study	Private periodontal practice	Patients diagnosed with chronic adult periodontitis, with each patient receiving surgical therapy in two or more quadrants. The group was divided into two subgroups: those who complied with the recommended maintenance schedule and those whose compliance was erratic	Mean number of years of the observation period was 6.7 years, with a range of 9 years (3–12 years)	92 patients (37 male and 75 female) with 2310 teeth no tooth loss: 66 patients (72%) 1–3 teeth lost: 23 patients (25%) more than 3 teeth lost: three patients (3%)	Tooth prognosis was determined from radiographic examination by: Severity of BL Presence or absence of angular defects Degree of FI		Clinical criteria tresholds	During the SPT	>5 years tooth survival	Good Questionable Hopeless	No AUC No specificity No sensitivity Patients complying erratically with supportive periodontal therapy were at a 5.6 times greater risk for tooth loss following active therapy than regularly compliant patients	Teeth lost (during the SPT)	Distribution-free’ tests (Fisher ex-act test, Chisquare, Mann–Whitney U-test). Unconditional logistic regression was used to calculate odd ratios (OR) and 95% conﬁdence intervals (CI). Stat- istical analysis was performed by using the SPSS-X statistical package (SPSS 1998)
Fardal et al (2004)^12^	Tooth	Retrospective study	Norwegian specialist periodontal practice	Patients who had comprehensive periodontal treatment	Attended for 9.8 (SD: 0.7), range: 9–11 years of maintenance care	100 consecutive patients (68 females, 32 males)	PPD Proximal BL FI BOP Mobility		Clinical criteria tresholds	After the completion of the active phase of periodontal treatment and prior to placing the patient on maintenance A prognosis was assigned to all teeth that were present after 9–11 years of maintenance care.		Good Uncertain prognosis Poor Hopeless	No AUC No specificity No sensitivity Patient level: risk increase for male (versus female); for smoker (versus non- or former smoker), for older age (>60 years versus younger)	Tooth lost	
Kwock and Canton (2007)^20^	Tooth		/	/	/	/	Patient compliance Smoking Diabetes Neutrophil dysfunction PPD CAL FI Enamel pearls Cervical enamel projections Tooth position Overhanging restorations Trauma from occlusion Parafunctional Habits Mobility		Diagram Clinical measures	Before treatment Prognosis can change after treatment as well as after recurrent disease activity. Therefore, reprognostication occurs after each examination of the patient.	Long-term prognosis for 5 years, but reassessment is often needed for a prolonged period	Favorable Questionable UnfavorableHopeless	No AUC No specificity No sensitivity	Probability of disease progression (with periodontal treatment and maintenance)	Prognostication system, based on stability and evidence-based modification factors
Faggion Jr et al (2007)^11^	Tooth	Retrospective study	Department of Periodontology, University of Munster, Germany	Periodontal patients with at least 5 years of SPT	Mean: 11.8 ± 2.3 years (range: 7.91–15.67 years)	198 patients4393 teeth; 249 teeth lost	Tooth mobility Tooth type Alveolar bone level Pulp vitality Diagnosis of diabetes		Graph	Estimates of tooth survival probability when periodontal therapy is rendered.	Tooth survival probability over an average of 11.8 years	Provides probability of tooth survival in 10% increments	NO AUC No specificity No sensitivity	Tooth loss (all causes) during SPT	Logistic regression
Avila et al (2009)^4^	Tooth		/	/	/	/	Factors and variables that can inﬂuence the ﬁnal decision to save or extract a tooth into six levels: 1) initial assessment 2) periodontal disease severity 3) furcation involvement 4) aetiologic factors 5) restorative factors 6) other determinant (smoking, biphosphonates, systemic conditions, clinician skills)		Color-based decision-making chart (green, yellow, and red)	Before taking the decision to save or extract a compromised tooth	Long-term survival	Red: long-term survival unfavourable Yellow: proceed with caution recommend Green: long-term maintenance favourable	No AUC No specificity No sensitivity This model, notably, was originally to be used as ‘decision chart’, supporting decision-making towards tooth retention or removal	Tooth loss, extraction vs conservation	Specialized scientiﬁc literature supporting the concepts proposed in our decision-making process was selected after performing a search in three databases (PubMed, Ovid, and Scopus) using MeSH and non-MeSH terms related to each category of the chart
Graetz et al (2011)^13^	Tooth	Longitudinal retrospective study	Department of Periodontology, Christian-Albrechts-University of Kiel, Germany	Patients with aggressive periodontitis (AgP) and chronic periodontitis (CP)	The total observational period was 16.1 4.5 (10–24) years for AgP patients and 16.3 4.1 (10–24) years for CP, respectively; the mean duration of the SPT was 15.3 4.1 years for AgP and 15.7 3.6 for CP	34 AgP and 34 CP patients (SPT>10 years)	BL		Two definitions : teeth were categorized as ‘questionable’ if they had lost from >50 to <70% of bone height at one proximal surface or ‘hopeless’ if they had lost >70%	Before and after active periodontal therapy (APT) and SPT	Accurate estimation 15 year survival	Questionable Hopeless	No AUC No specificity No sensitivity	Tooth loss (SPT)	
Nunn et al (2012)^33^	Tooth	Retrospective study	Private periodontal practice	Periodontitis patients with at least 5 years of SPT	Mean: 9.97 years (range: 0.33 – 15.17 years)	100 patients2509 teeth; 131 teeth lost	PPD FI Root form C/R ratio Mobility BL Root proximity Hygiene level Tooth malposition Fixed abutment status Removable abutment status Bite guard Parafunctional habit Percentage BL Compliance Family periodontal history		Multivariate exponential survival trees in terms of molars and non-molars	After SPT	Accurate estimation 15 year survival	Good Fair Poor Questionable Hopeless	‘Sensitivity and specificity are relatively high’	Tooth loss (all causes)	Classification and Regression Trees
Miller et al (2014)^27^	Tooth (molar)	Retrospective study	Private periodontal practice from 1969 to 1994	Moderate to severe periodontitis patients with at least 15 years of SPT all ﬁrst and second molars present at the initial examinatio	>15 years (no mean/SD reported)	102 patients816 molars; 177 teeth lost	Age PPD Mobility FI Molar type Smoking		Numeric score Scoring index	Before periodontal treatment	Accurate estimation of the 5‒10, 10‒20, and 20‒30 year survival	Provides a score between 1-11 Initially assigned their scores to prognostic factors specific to a tooth type (molar teeth) to derive a quantitative scoring system	NO AUC No specificity No sensitivity	Tooth loss from any cause (including during IPT)	Cox proportional hazards model with robust sandwich variance estimate to account for clustering ⁺
Niballi et al (2017)^32^	Tooth	Retrospective study	UK private practice setting	Periodontitis patients, with at least 5 years of SPT with at least 1 session of SPT every year	Mean: 79.1 ± 18.0 months (range: 60 – 123 months)	98 patients; 23 patients lost teeth 2395 teeth; 34 teeth lost	PPD FI Mobility BL Periapical pathology (periapical index, PAI, score of 5 Restorability		Diagram	Before active therapy	>5 years	Good Fair Questionable Unfavorable	NO AUC No specificity No sensitivity	Tooth loss (all causes) during SPT	Logistic regression; Poisson regression
McGowan et al (2017)^25^	Tooth		/	/	/	/	9 evidence-based quantifiable parameters Six tooth-level risk predictors (BL:age, PPD, FI, infrabony defects, anatomical factors, and mobility) and 3 patient-level modifiers (smoking, diabetes, and BOP)		Evidence-based prognostic model Table with treatment recommendations	At the time of initial assessment although ideally the formal prognosis should be delayed until after completion of the active phase of peri- odontal treatment to improve accuracy.	Either short (<5 years) or long (>5 years) term	Secure Doubtful Poor Irrational to treat	NO AUC No specificity No sensitivity	Used at the time of initial assessment if the patient requests some indication of expected outcome or to determine which teeth are irrational to treat, although ideally the formal prognosis should be delayed until after completion of the active phase of periodontal treatment to improve accuracy	
Martinez-Canut et al (2018)^24^	Tooth	Retrospective study	Private periodontal practice in Valencia, Spain	Periodontitis patients with at least 17 years of SPT, with at least one visit per year for SPT	Mean: 20.2 years (±2.4), ranging from 17 to 25 years	500 patients12830 teeth; 515 teeth lost due to periodontitis	Age, severe periodontitis, heavy smoking, bruxism, baseline number of teeth; type of tooth, FI, PPD, BL, mobility, C/R ratio		Web- based algorithm (www.perioproject.es) Long-Term Outcome (LTO) index			Predicting TLPD+ or TLPD–	Discrimination measurements (for molars and non- molars, respectively) were as follows: AUC 0.93 and 0.97; sensitivity 39% and 43%; specificity 98% and 99%	Tooth loss due to periodontitis	Generalised linear mixed multilevel regression (logit link function, using a binomial distribution)
Santos et al (2021)^49^	Tooth	Retrospective study develop and validate a predictive model	Periodontology Department of Egas Moniz Dental Clinic (EMDC)	Periodontitis patients	Between May 2015 and March 2020	455 patients were eligible for the model development, while the remaining 99 individuals were used for validation purposes	Tooth type Clinical attachment loss		multivariable model	Only teeth extracted due to periodontal reasons and before the beginning of the non-surgical periodontal treatment were included		Equation modelling tooth loss prediction (extraction by surgery), in periodontitis patients, could be identified as: log [Probability (extraction)/(1 − Probability (extraction))] = −7.850 + (0.589 × incisor) + (0.661 × CAL)	Development : AUC = 0,920 Validation : AUC = 0,809	early tooth loss due to periodontitis	
Rahim-Wöstelfeld et al (2022)^40^	Tooth	Explorative analysis	University hospital in Heidelberg	Periodontitis patients	Mean: 10 years ± 6 months	110 patients2528 teeth; 166 teeth lost	Age (years) Gender PCR Smoking Diabetes Jaw Tooth type FI Interproximal BL Infrabony defect Abutment tooth		Tree model	At the beginning of the active periodontal therapy	10 years after APT	8 categories	AUC of 0.77 Sensitivity of 0.73 (95% CI [0.64; 0.80]) Specificity of 0.79 (95% CI [0.70; 0.86])	Tooth loss (all causes)	Generalised linear mixed model tree
Saleh et al (2022)^47^	Tooth	Retrospective study	University setting	Periodontitis patients, with at least 10 years of SPT, at least 1 SPT session per year, whose teeth were extracted at the university	Mean: 22.6 years (range: 10–47.6 years)	340 patients7924 teeth; 303 teeth lost due to periodontitis	Age Smoking A1c levels Tooth type Mobility PPD FI		Modified Miller‒McEntire Periodontal Prognostic Index (MMPPI) Numeric score Scoring index	Before periodontal treatment	Accurate estimation of the 5‒10, 10‒20, and 20‒30 year survival	Class 1 excellent Class 2 good Class 3 guarded	AUC of 0.7 Moderate sensitivity (78.05%) and speci- ficity (69.60%)	Tooth loss due to periodontitis	Multilevel Cox frailty regression


**Table 2:** External validations

Author (year)	Type of study	Setting	Population characteristics	Prognostic tool evaluation	Main results
Schwendicke et al (2018)^53^	Retrospective study	University setting	301 periodontitis patients active periodontal therapy SPT in a university setting Follow-up 21.7 ± 5.6 years	Avila et al (2009) AUC = 0.67 Faggion et al (2007) AUC = 0.57 Nunn et al (2012) AUC = 0.64 Fardal et al (2004) AUC = 0.62 Pretzl et al AUC = 0.57 (0.55/0.59)	The best predictive value was found for the model by Avila et al (2009) In general, most models overestimated the risk of TL, that is, when applying the models at more sensitive cut-offs, weoften predicted TL for teeth which were eventually retained.
Saleh et al (2021)^46^	Retrospective study	University of Michigan School of Dentistry, Ann Arbor, MI, USA	148 patients with 3,787 teeth followed-up for a mean period of 26.5 ± 7.4 years	Grant et al (1979) Becker et al (1984) McGuire (1991) Checchi et al (2002) Fardal et al (2004) Faggion et al (2007) Kwok & Caton (2007) Avila et al (2009) Nunn et al (2012) Miller et al (2014)	All included systemsaccurately stratified teeth based on the risk of TLP at baseline whendifferent classes of association were compared. Most of the classes of all prognostic systems showed statistically significantinter-class differences. In most prognostic systems, the moresevere classes were associated with a higher risk of TLP Miller and McEntire system may have thehighest predictive capacity, which showed the best values in terms of model performance and fit after both univariate and multivariate analysis
Saydzai et al (2022)^51^	Retrospective study	Three different private clinics in London and Hertfordshire (United Kingdom), after being referred by their general dental practitioner	97 patients Regular SPT for 5 years	McGuire & Nunn (1996) Kwok & Caton (2007) Graetz et al (2011) Nibali et al (2017)	All four systems showed good reproducibility and could identify teeth at higher risk of being lost during 5 years of SPT The risk of TL increased with the worsening of tooth-prognosis category (p <.0001). Although specificity and negative predictive values were good, low sensitivity and positive predictive values were detected for all systems


Traditionally, classical prognosis systems identified teeth that were at high risk of being lost. Hirschfeld and Wasserman and Becker categorised teeth into ‘favourable/questionable’ and ‘questionable/hopeless’ prognosis categories, respectively, based on tooth-related factors such as FI, PPD, BL and degrees of mobility.^5,17^ Checchi et al (2002), in a cohort of 92 patients with 2,310 teeth, distinguished ‘good/questionable/or hopeless’ teeth based on tooth-related factors (severity of BL).^7^ These studies conclude that teeth with severe periodontal BL (>75 %) have the highest risk of being lost after 5 years. Patients complying erratically with SPT were at a 5.6 times greater risk for TL following active therapy than regularly compliant patients.^7^ In these prognostic tools, patient-related factors were not considered.

Kwok and Caton (2007) considered general (compliance, plaque accumulation, smoking, diabetes, and others) and local factors (PPD, attachment loss, anatomic plaque-retentive factors, trauma from occlusion and habits, mobility) based on literature evidence.^20^ Individual tooth prognosis is based on the prediction of future stability of the periodontal supporting tissues with four classes (favourable, questionable, unfavourable, hopeless).

Graetz et al (2011) conducted a study comparing patients with ‘aggressive’ (34 patients) and ‘chronic’ (34 patients) periodontitis, assigning their prognosis of TL based on just the extent of BL alone with a two-category system, showing increasing TL rates for ‘hopeless’ compared with ‘questionable’ teeth.^13^ With a periodontal therapy and a stringent maintenance programme, 88.2% of the questionable teeth and 59.5% of the hopeless teeth survived over 15 years of SPT.

The retention of a periodontally compromised tooth, as opposed to tooth extraction followed by prosthetic replacement, is a complex, multifactorial decision that clinicians must take. The aim of periodontal therapy is to preserve teeth as long as possible in function and acceptable aesthetics. Avila et al (2009) elaborated a colour-based decision-making chart (green, yellow, red) to guide clinicians in making the decision to conserve or extract a compromised tooth.^4^ Factors that can influence the final decision to save or extract a tooth were divided into six levels: initial assessment, periodontal disease severity (PPD, mobility), furcation involvement, aetiologic factors (presence of calculus, root anomalies), restorative factors (extensive caries, crown/root ratio (C/R ratio)) and other determinants (smoking, systemic conditions). The chart should be interpreted level by level, starting at level one and continuing to level six. The green category suggests the favourable long-term conservation of the tooth, the yellow means that saving the tooth depends on the control or suppression of some modifiable factors, and the red indicates an unfavourable long-term outcome of the tooth.

McGowan et al (2017) elaborated an evidence-based prognostic model to give each tooth a prognosis of secure, doubtful, poor, or irrational to treat.^25^ It provides short- (5 years) and long-term (10 years) clinical expectations with specific recommendations for interdisciplinary treatment (endodontic, prosthodontic, and restorative). Six tooth-level (BL/age-ratio, PPD, extent of FI, infrabony defect, compromising anatomical factors, extent of mobility) and three patient-level (smoking, poorly controlled diabetes, BOP) factors are evaluated. If one or more of these patient-level factors are present, the tooth-level assessment is downgraded by one level to a maximum of poor.

Using the Cox hazards regression model, McGuire and Nunn proposed a periodontal prognostic system in 1996 and tested its association with TL in a cohort of 100 treated periodontal patients (2,484 teeth) under SPT for 5 years, with 38 of these patients followed for 8 years.^26^ Teeth were classified into five categories: good, fair, poor, questionable, and hopeless. Key clinical parameters were identified with significant association for TL, like PPD (regression ratio RR = 1.39), FI (RR = 1.29), mobility (RR = 2.05), percentage of bone loss (RR = 1.04), parafunctional habit without a bite guard (RR = 2.17), and smoking (RR = 2.06).

Based on clinical and radiographic ﬁndings (PPD proximal BL, FI; BOP, mobility) using a multivariable logistic regression analysis, Fardal et al (2004) assigned to each tooth a prognosis (good, uncertain, poor, or hopeless) after the completion of the active phase of periodontal treatment and prior to placing the patient in SPT. Male gender (OR = 2.8), age >60 years (OR = 4.0), and smoking (OR = 4.2) were identified as signiﬁcant predictors of TL by logistic regression analysis. This logistic regression model also included non-signiﬁcant variables such as frequency of maintenance, oral health status, and family history of periodontal disease as independent variables. Compliance with maintenance following periodontal treatment was associated with very low levels of TL. Only 36 (1.5%) of the 2436 teeth present at baseline were subsequently lost due to periodontal disease.

Using a logistic regression model, Faggion et al (2007) developed a prognostic model to estimate survival rates of teeth in periodontally compromised patients over an observation period of 11.8 years.^11^ A generalised linear model was established and a graph was constructed over the full range of all variables with signiﬁcant prognostic value. Variables such as diabetes and BL (additionally, tooth mobility, and root type) were identified as influential predictors for TL. The prognostic model demonstrates that the risk increases for patients with diabetes mellitus (systemic diseases) compared to those without (OR = 4.17). At the tooth level, the risk increases for each percentage of BL (OR = 1.04), for mobility III (versus all others OR = 5.52), for multi-rooted (versus single-rooted OR = 1.82) and for root-canal treatment (OR = 2.24). For example, multi-rooted, vital teeth in non-diabetic patients with a BL of 40% have a probability of 80–89% tooth survival, while in contrast, diabetic patients with a BL of 40% showed a 50–59% probability of tooth survival over an average of 11.8 years.

From 100 patients (2509 teeth) in maintenance, initially diagnosed with moderate to severe periodontal disease, Nunn et al (2012) elaborated two multivariate exponential survival trees in terms of molars and non-molars based on tooth (PPD, FI, root form, BL) and patient factors (compliance, family periodontal history, parafunctional habit).^33^

Miller et al (2014) proposed a prognostic model only for molars, the Miller‒McEntire Periodontal Prognostic Index (MMPPI), which provides a score between 1 and 11.^27^ The variables influencing the score are molar type (maxilla/mandibula/first or second molar) (HR = 1.20), smoking (HR = 3.46), FI (HR = 1.21), PPD (HR = 2.20), mobility (HR = 2.08), age (HR = 1.27) and diabetes. A statistically derived score was determined for each factor. The sum of these scores became the score for that tooth. There is a 38% increase in risk for molar extraction with every unit increase in score (HR = 1.38). This index has undergone several modifications to accommodate the significance of environmental factors.^21^ powerful, evidenced-based, statistically validated, and accurate motivational tool which can be used daily in clinical practice with all patients. Its usage is not limited to patients presenting with periodontitis but is routinely used with periodontally healthy patients. In other words, the MMPPI can be used for all patients. The benefits to the patient are that they better understand their long-term periodontal prognosis of 15 and 30 years. It also encourages patients to make lifestyle changes to improve their overall health including smoking cessation, blood sugar control, and compliance to their recommended preventative periodontal maintenance frequency. Accurate prognosis can be determined by scoring the most periodontally involved molar that you plan to keep. The strength of the MMPPI is that it translates clinical outcomes into patient value.” ,”ISBN”:”978-3-030-12309-3”,”note”:”DOI: 10.1007/978-3-030-12310-9_1”, ”page”:”3-21”,”source”:”ResearchGate”,”title”:”The Miller McEntire Periodontal Prognostic Index (i.e., “The Perio Report Card” More recently, in 2022, Saleh et al proposed a modified version of the MMPPI, called the periodontal risk score (PRS). Instead of 11 classes, this modification has only three (1 = excellent prognosis; 2 = good prognosis; and 3 = guarded prognosis), and it can be applicable for anterior as well as posterior teeth. Haemoglobin A1c (HbA1c) levels instead of binary (diabetic/non-diabetic) records were required. Cigarette consumption (smoker/non-smoker) was changed into never, former, current light, or current heavy smokers. Both scores allow for an accurate estimation of the 5‒10, 10‒20, and 20‒30-year tooth survival rates. The PRS showed moderate sensitivity (78.05%) and specificity (69.60%).

Nibali et al (2017) proposed a prognostic tool to assess the risk for TL in periodontally compromised patients at the beginning of treatment in order to support treatment planning.^32^ Logistic regression was used to evaluate the associations between the incidence of tooth loss and potential risk factors. Teeth were assigned either a good, fair, questionable, or unfavourable prognosis based on variables adapted from other studies^5,13,23,26^ such as PPD, FI, mobility, BL, but also non-periodontal factors such as periapical pathology and restorability. A strong association was detected between this prognosis system and TL during SPT.

Martinez-Canut et al (2018) developed two separate models (molars and non-molars) in the form of a web-based algorithm to assign periodontal prognosis based on survival time in 500 patients following periodontal maintenance.24 The tool calculates the probability of TL due to periodontal disease according to the impact of 11 predictors, and this probability can be associated with a certain survival time. The model presented high specificity and moderate sensitivity. Discrimination measurements for molars and non-molars were, respectively, AUC 0.93 and 0.97; sensitivity 39% and 43%; specificity 98% and 99%.

In two different cohorts, Santos et al (2021) developed and retrospectively validated a predictive early TL multivariable model for periodontitis patients before periodontal treatment. Only teeth extracted due to periodontal reasons and before the beginning of the non-surgical periodontal treatment were included.^49^ The factors ‘tooth type (incisor)’ and CAL were identified as significant predictors (P = 0.037 and P <0.001, respectively). An equation modelling TL prediction was proposed based on these parameters; the AUC was 0.809 (95% CI: 0.629–0.989) for the validation sample and 0.920 (95% CI: 0.891–0.950) for the development cohort.

In the study by Rahim-Wöstefeld et al (2022), a generalised linear mixed model tree was used to develop a prognostic model for TL based on 110 periodontally treated patients with 2,528 teeth 10 years after active periodontal therapy.^40^ It takes into consideration variables at both patient-level (age, diabetes) and tooth-level as FI (single-rooted/multirooted without/with FI), interproximal BL and abutment tooth (no/fixed/removable dentures). Abutment teeth (fixed or removable) with a periodontal BL ≤ 60%, being single-rooted or multi-rooted without FI in patients >61 years of age, are at the highest risk for TL (50.0%). The prediction model reached an AUC of 0.77.

In a recent retrospective study, Saleh et al (2021) externally validated 10 commonly used tooth-level prognostic systems in 148 patients with 3,787 teeth regarding their predictive capacity for the risk of TLP.^46^ All included systems accurately stratified teeth based on the risk of TLP at baseline when different classes of association were compared. Most classes across all prognostic systems showed statistically significant inter-class differences. In accordance with the study by Saydzai et al (2022), which evaluated four different periodontal prognostic systems, the worst tooth-prognosis classes were associated with a higher risk of TL.^51^ In general, most models overestimated the risk of TL; when applying the models at more sensitive cut-offs, TL was predicted for teeth which were eventually retained. Although specificity and negative predictive values were good, sensitivity and positive predictive values were low across all systems, ranging from 3% to 12%.^51^ Most models showed low accuracy (AUC ranged between 0.52 and 0.67).^53^ Know and Caton et al (2007), Nunn et al (2012), and Fardal et al (2004) were externally validated.^49,54,56^

Recently, AI has emerged as a promising tool for predicting and managing various diseases.^52^ AI refers to computer systems that simulate intelligent human behaviour. In periodontology, AI has been used for risk prediction, diagnosis, and treatment planning.^61^ AI techniques such as machine learning, neural networks, and deep learning have been used to analyse large data sets and identify patterns and correlations that may be missed by traditional methods (Table 3).^54^ It has the potential to improve the accuracy of risk assessment and provide personalised treatment planning for patients. Troiano et al (2023) developed and validated models based on logistic regression and artificial intelligence for prognostic prediction of molar survival in periodontally affected patients.^61^ The combination of neural network and logistic regression showed the best performance for the prediction of molar loss with an AUC of 0.726. In the development and external validation study of Santamaria et al (2024) from two different clinical centres, the traditional tool of Nibali et al (2017) was compared with an AI-based model for tooth prognosis in patients with periodontitis in SPT for 10 years.^48^ A periodontal prediction model was developed using a neural network with the following parameters: PPD, mobility, FI, %BL and PA. AI-based models showed comparable results with the clinical prediction model, with the same value of area under the curve (AUC) = 0.8943. These observations confirm that the AI prediction model is a promising tool for the prediction of TL.

**Table 3:** Artificial intelligence models

Author (year)	Type of study	Setting	Population characteristics	Prognostic tool evaluation	Main results
Troiano et al (2022)^61^	Retrospective study Developpement and external validation	Four different universities (two in Europe, onein the United States, and one in China)	515 patients and 3157 molars Prognostic ability of molar loss over 10 years	Logistic regression and artificial intelligence for prognostic prediction of molar survival in periodontally affected patients Logistic regression Support vector machine K-nearest neighbours Decision tree Random forest Artificial neural network Gradient Boosting Naive bayes	Ensembled model, which combined neural network and logistic regression, showed the best performance among the different models for the prediction of MLO with an area under the curve (AUC) = 0.726. The neural network model showed the best AUC of 0.724 for the prediction of periodontitis-related molar loss. In addition, the ensembled model showed the best calibration performance
Santamaria et al (2024)^48^	Retrospective study	2 different clinicalcentres : a private clinic in London (United Kingdom) and the peri-odontology department of the University of Pittsburgh (USA)	1626 teeth in 69 patients were included in the London cohort 2792 teeth in 116 patients were included in the Pittsburgh cohort SPT for 10 years	Nibali et al (2017) Neural network model (NN) Showed the same value of area under the curve (AUC) = 0.8943 TPS in the validation cohort exhibited high specificity (99.96%), moderate positive predictive value (PPV = 50.0%) and very low sensitivity (0.85%) AI-based model showed moderate specificity (NN = 52.26%, LR = 67.59%), high sensitivity (NN = 98.29%, LR = 91.45%), and high PPV (NN = 89.1%, LR = 88.6%)	AI-based models showed comparable results with the clinical prediction model, with a better performance in specific prognostic risk categories, confirming AI prediction model as a promising tool for the prediction of TL


## DISCUSSION

### Main Findings and Quality of Evidence

The determination of prognosis is an integral part of periodontal practice and an evolving and dynamic process. A clinically relevant periodontal prognosis model must be able to accurately predict the course of disease in a way that has meaning to both the patient and the clinician. It should be based on the best available evidence and use well-defined, quantifiable parameters that can be accurately recorded or collected. It helps to establish a realistic treatment plan and limit the financial implications of complex periodontal disease. It is commonly expressed as either short (<5 years) or long (>5 years) term.^20^ Different models have been described to establish the clinical limits between treatable and untreatable conditions. These tools are based on prognostic factors which can be categorized as: (1) those that can be controlled by the patient (daily plaque removal, smoking cessation, compliance with wearing occlusal guards, compliance with the recommended preventive maintenance schedule); (2) those affected by periodontal treatment (PPD, mobility, FI, trauma from occlusion, bruxism, other parafunctional habits); (3) those associated with systemic diseases (diabetes mellitus, immunologic disorders, hypothyroidism); and (4) those that are uncontrollable (poor root form, poor C/R ratio, tooth type, age, genetics).^30^ Stage III or stage IV periodontitis, age, gender, molar teeth, PPD, BL, the presence of a root-canal treatment and a crown are prognostic factors associated with TL.^1^ When clinical parameters and risk factors are evaluated (individually or in combination), many confounding factors can influence long-term tooth retention.^14^ The determination of prognosis can become more scientific by incorporating evidence-based modification factors on disease progression. In addition to tooth-related parameters, patient-related factors are strongly associated with TL and need to be considered.

At the initial examination, prognosis is determined based on the patient’s initial status, treatment expectations, and the uncertainty of controlling certain prognostic factors. Patients are educated and motivated on factors that can be changed during the treatment. At the re-evaluation visit, prognosis is determined again because some factors may have changed, or new findings may have emerged during therapy. Therefore, treatment plans may be reviewed along with further patient education. Finally, after comprehensive periodontal treatment, the prognosis is reviewed again based on the results, and the necessity for future treatment needs can be discussed.^3^

The utilisation of prognosis tools in patient care settings must be guided by evidence. The specificity is the capability of a tool to identify a tooth with a good prognosis. The sensitivity is the ability to identify a tooth with a hopeless prognosis. The higher the values of a test’s sensitivity and specificity (each out of 100%), the more accurate the test is in diagnosing a disease or condition.^55^ Most prognostic models confirm high precision and accuracy, fluctuating in most cases around a value of AUC = 0.8. It was demonstrated that the complexity and the number of factors included in the prognostic system do not necessarily affect accuracy.^19,39^ McGuire and Nunn (1996) observed that it is difﬁcult to accurately assess the correct prognosis for teeth affected by periodontitis as there are many clinical factors which must be taken into consideration.^26^ In addition, descriptive terms such as good, fair, or questionable can be perceived subjectively by clinicians due to the heterogeneity in the interpretations and applications of these definitions.^11,20,27^

Even though many of the prognosis models have shown promising results in the populations they were originally developed in, the external validity of prior findings remains to be tested on a new cohort.^20,53^ Such validation is important because of the lack of evidence to determine whether tooth-based risk assessment systems assign a meaningful prognosis capable of accurately predicting long-term outcomes and tooth survival. Due to the complexity of periodontal disease and factors such as systemic conditions and dentist expertise, there is no universally accepted standard for periodontal prognosis. Many models predicting TL in periodontitis are available, but studies evaluating these models are at a high risk of bias. Our results are in agreement with the systematic review of Chow et al that included prognosis tools at the tooth but also at the patient level.^9^ While both reviews are unable to recommend any model for clinical practice, the existing models and their model performance at external validation were synthesised, which would be helpful to identify promising models for future external validation studies.

The definition of hopeless teeth has not evolved from the classical points based on the severity of disease.^5,26^ Severe bone loss, deep PPDs, FI, tooth mobility, unfavourable C/R ratio, root proximity or presence of abscess have been used to categorise teeth as hopeless. According to the recent guidelines, it is crucial to distinguish between questionable and hopeless teeth, as early extraction of questionable teeth is discouraged, and only ‘hopeless’ teeth should be extracted.^50^ The classification model proposed by Avila et al (2009), expressing the risk of TL in five grades, takes into consideration the strategic value of a tooth in the treatment plan.^4^ The McGowan’s tool attributes to a tooth a prognosis of secure, doubtful, poor, or irrational to treat.^25^ It provides indications for additional (endodontic, prosthodontic, and restorative) interdisciplinary treatment. In the era of classical periodontology, success was regarded as the resolution of the disease, including strategic extractions to achieve a mean PPD reduction. The current definition of success is best expressed as an effort towards the preservation of natural dentition associated with patients’ well‐being.^37^ Tooth extraction should only be considered in cases of patient discomfort, concern for the patient’s general health, and documented loss of attachment of adjacent teeth. Despite extraction being proposed as the treatment of choice, 77% of non‐extracted hopeless teeth were retained after a mean period of 5.25 years without loss of attachment of the adjacent sites.^5,23^ Even teeth with an initial bone loss of over 60% could be retained in approximately two-thirds for 20 years. Prognoses often changed over time, and changed most frequently in teeth that had less than ‘good’ prognoses initially.^26^ This should be kept in mind when assigning prognosis and establishing a treatment plan. The ability to preserve compromised teeth through powerful treatment modalities able to change tooth prognosis is a critical component of modern dentistry, as it makes the goal of lifelong preservation of function possible even for subjects with severe conditions such as periodontitis. Periodontal therapy and SPT are effective in maintaining most of the teeth in patients with moderate/advanced periodontitis for 30 years.^1^ Adherence to SPT should be strongly promoted, since it is crucial for long-term periodontal stability, potential further improvements in periodontal status and oral-health-related quality of life.^36,42,50^ The mean prevalence of TL in patients in SPT for 5 years or more is less than 10%, with a tendency for greater prevalence with time. A successful treatment plan should always evaluate the potential effectiveness of regeneration therapy before opting for any extractions. Periodontal therapy can change the prognosis of hopeless teeth, making them maintainable in the long term.^37^ A study of Cortellini et al (2020) compared clinical- and patient-based outcomes following periodontal regeneration or extraction and replacement of hopeless teeth with chronic perio-endo lesions and/or attachment loss to or beyond the apex.^10^ The 10-year survival of regenerated teeth was 88%, with no statistically significant difference comparing the survival of implant- or tooth-retained fixed partial dentures. The rate of TL can be minimised in a way that a period of 10 years or more is needed to evaluate further periodontal breakdown. In addition, patients’ perception and satisfaction with the treatment should be considered as the main therapeutic endpoints of the provided periodontal therapy.

Tooth loss can result from clinical, iatrogenic, or natural causes and, therefore, may not serve as an ideal prognostic indicator.^25^ Consequently, periodontal stability has been suggested to offer greater prognostic value and to be more relevant in clinical practice. The main criticisms of existing systems include subjectivity, complexity, and the failure to account for restorative or patient-related factors.^25^ It is acknowledged that no guideline for determining whether to extract or preserve a compromised tooth can be entirely flawless. Ultimately, the clinician holds the responsibility for making the final decision. A comprehensive assessment of all relevant factors is essential, as no universal rule applies to every case.^34^

### Implications for Practice

The idea is to help practitioners identify teeth that appear to have a more favourable treatment outcome, as opposed to those more severely compromised by disease. Although assigning prognosis is primordial in clinical practice, no clear guidelines have yet been established. Any existing prognosis tool can be clearly recommended. An efficient prognosis tool should include selective predictive factors, their assigned weights in accordance on different tooth conditions, and the estimated period for re-evaluation of prognosis. It needs to be quick and easy to use and requires information that is available to clinicians.^60^ One main disadvantage of some tools in this study is the time and the complexity of the parameters required to execute the prognostication.

### Implications for Research

A predictive model that is built on retrospective data should be validated prospectively in future studies with a low risk of bias. Machine learning is an emerging approach to prediction models. Future research comparing machine-learning approaches with traditional regression-based approaches should be developed. Integrating AI into healthcare has revolutionised diagnostic and treatment approaches in many fields, and dentistry is no exception.^38^ AI models for periodontology applications are still in development but might provide a powerful diagnostic tool capable of indicating the rate of future TL.^44^ Machine learning works by using algorithms that allow a computer system to learn from data, recognise patterns, and make decisions without prior programming. The recognition process is therefore based on learning from selected data rather than predefined algorithmic programming. Machine learning requires a large amount of training data to produce accurate and reliable results. The better the data, the more accurate the model will be.

The use of AI in healthcare may be hindered, however, by issues like multidimensional integration of information, safety, federated learning (which necessitates essential developments in fields like privacy, large-scale artificial intelligence, and distributed optimisation), model performance, and bias.^58^ One of the main problems is that most studies investigating the use of AI in periodontology are based on limited and unrepresentative data sets with a high risk of bias. The systematic review of Hasuike et al (2022) included five studies with high AUC (>0.8) and accuracy, however, they were broadly assessed to have a high risk of bias.^15^ The ‘blackbox’ problem in AI models used in periodontology is linked to the difficulty of understanding how the models made their decisions.^8^ Indeed, AI models can be very complex and include many computational layers, making them difficult for humans to understand. This opacity can be detrimental in a field such as periodontology, where healthcare professionals need to understand how a model made its decisions to validate and use them with confidence. Furthermore, a lack of transparency in AI models can also raise ethical issues. Medical and dental data are not as available and accessible as other data, due to data protection. The single information provided by most of today’s dental AI applications will only partially inform the required and complex decision-making in clinical care.^52,58^ If an AI model is used to make treatment decisions for a patient, it is important that the patient understands how these decisions were made and trusts their validity. If AI models are opaque and difficult to understand, this can create a feeling of misunderstanding or mistrust among patients. Questions towards responsibilities and transparency remain.^52^

### Limitations of the Study

The present study has some potential limitations. Most of the studies have a retrospective design with a high risk of bias. The populations included in these studies are very specific, with patients treated for periodontitis and compliant during all the follow-up, which limits generalisability to other populations. In addition, only prognostic tools at the level of the tooth were included. Restriction to tooth-level prognostic tools excludes broader patient-level models, which might also be relevant.

## CONCLUSION

A clinically relevant periodontal prognosis model must be able to accurately predict the course of disease in a manner that is meaningful to both the clinician and the patient. All prognostic systems presented a very good capacity to predict TL linked to periodontal disease. Although AI-based tools have the potential to significantly improve periodontal disease management, their use must be approached with caution. Particular attention and methodological guidance are needed to improve the quality of machine-learning-based clinical prediction models. Legitimate concerns exist regarding data protection, data security, and the reliance on computer-based systems for critical medical decision-making. Further studies are needed to evaluate their effectiveness in larger, more representative datasets and to ensure their use is effectively integrated into personalised patient care.
